# An Automated Approach to Examining Conversational Dynamics between People with Dementia and Their Carers

**DOI:** 10.1371/journal.pone.0144327

**Published:** 2015-12-10

**Authors:** Christina Atay, Erin R. Conway, Daniel Angus, Janet Wiles, Rosemary Baker, Helen J. Chenery

**Affiliations:** 1 Asia-Pacific Centre for Neuromodulation, UQ Centre for Clinical Research, Faculty of Medicine and Biomedical Sciences, The University of Queensland, Herston, Queensland, Australia; 2 School of Allied Health, Faculty of Health Sciences, Australian Catholic University, Banyo, Queensland, Australia; 3 School of Communication and Arts, Faculty of Humanities and Social Sciences, The University of Queensland, St Lucia, Queensland, Australia; 4 School of Information Technology and Electrical Engineering, Faculty of Engineering, Architecture and Information Technology, The University of Queensland, St Lucia, Queensland, Australia; 5 UQ Centre for Clinical Research, Faculty of Medicine and Biomedical Sciences, Herston, Queensland, Australia; 6 Faculty of Health Sciences and Medicine, Bond University, Robina, Queensland, Australia; Cardiff University, UNITED KINGDOM

## Abstract

The progressive neuropathology involved in dementia frequently causes a gradual decline in communication skills. Communication partners who are unaware of the specific communication problems faced by people with dementia (PWD) can inadvertently challenge their conversation partner, leading to distress and a reduced flow of information between speakers. Previous research has produced an extensive literature base recommending strategies to facilitate conversational engagement in dementia. However, empirical evidence for the beneficial effects of these strategies on conversational dynamics is sparse. This study uses a time-efficient computational discourse analysis tool called Discursis to examine the link between specific communication behaviours and content-based conversational engagement in 20 conversations between PWD living in residential aged-care facilities and care staff members. Conversations analysed here were baseline conversations recorded before staff members underwent communication training. Care staff members spontaneously exhibited a wide range of facilitative and non-facilitative communication behaviours, which were coded for analysis of conversation dynamics within these baseline conversations. A hybrid approach combining manual coding and automated Discursis metric analysis provides two sets of novel insights. Firstly, this study revealed nine communication behaviours that, if used by the care staff member in a given turn, significantly increased the appearance of subsequent content-based engagement in the conversation by PWD. Secondly, the current findings reveal alignment between human- and computer-generated labelling of communication behaviour for 8 out of the total 22 behaviours under investigation. The approach demonstrated in this study provides an empirical procedure for the detailed evaluation of content-based conversational engagement associated with specific communication behaviours.

## Introduction

Dementia is a common and significant health condition. Within the last 13 years, the number of people living with dementia worldwide has doubled from an estimated 24.3 million people in 2006 [1] to 47.5 million people reported in 2015 [2]. In addition to impaired memory functioning [3–6], one of the hallmark characteristics of dementia is a progressive decline in language skills [7–14] contributing to reduced quality of life in people with dementia and increased carer stress and burden [12, 13, 15, 16]. As the condition progresses, the main communication partners for people with dementia are typically their family and carers at home or residential aged care facility staff, with the overall opportunity for conversations being reduced [17]. Therefore, people with dementia commonly face a dual disadvantage of declining language skills coupled with a lack of spontaneous opportunities for conversational interaction [18], which puts them at risk for social isolation.

Engagement in meaningful conversations is important to the wellbeing of both people with dementia and their caregivers, but people involved in the care of people with dementia often require instruction and feedback to elicit and maintain those conversations [17]. To enhance the quality of life of people with dementia and quality of care, several publications have focussed on providing strategies for the improvement of communication between people with dementia and their conversation partners (see [16, 19, 20] for reviews). One recently published set of strategies is the MESSAGE training programme developed by communication experts at The University of Queensland, Australia [21–23].

### Recommended communication behaviours in dementia care

Informed by research findings on the specific communication support needs of people with dementia, the MESSAGE programme outlines communication strategies and specific communication behaviours for carers that are suggested to encourage conversational engagement with the person with dementia as well as preventing conversational breakdowns [23]. Two previous investigations have highlighted that the MESSAGE programme, together with its accompanying strategy set for memory support (RECAPS), was deemed a highly useful and applicable training tool by nursing home care staff [24], and resulted in increased perceptions of positive aspects of care-giving in training recipients [25]. Notwithstanding the favourable perceptions of the programme among care staff, it has not been empirically verified to date whether the spontaneous use of these recommended communication behaviours measurably facilitates conversational engagement between people with dementia and their conversation partners.

Therefore, the current investigation set out to examine whether spontaneous, pre-training instances of the use of communication behaviours that are representative of the MESSAGE strategies co-occur with instances of content-based conversational engagement. ‘Content-based conversational engagement’ in this investigation is defined as instances in a conversation where the same terms are being shared between speakers, as opposed to instances of no content-based conversational engagement where two conversation partners talk to each other without engaging with each other’s content. In an effort to generate replicable, objective data to evaluate content-based conversational engagement in the current study, Discursis [26, 27], a computer-assisted tool for discourse analysis, was employed in conjunction with traditional transcript coding techniques.

### Computer-assisted methods for discourse analysis

The analysis of real-life communicative interactions between humans is an integral aspect of research across numerous academic and clinical disciplines [28–34]. Irrespective of the discipline at hand, conventional discourse analysis techniques typically rely on human coders to qualitatively or quantitatively assess a given conversation within the theoretical framework of choice. Therefore, discourse analysis has long been inextricably linked to time-consuming, taxing work, with the common additional limitation of poor generalizability of findings outside a given analytical paradigm [35].

The advent of computer-assisted qualitative data analysis software (CAQDAS) provides a novel powerful approach for automating the previously labour-intensive process of detecting, labelling and quantifying discourse behaviour of interest. Apart from offering a relief from the burden associated with manual coding, CAQDAS-based discourse analyses can deliver rapid, replicable and objective output for the quantification of conversational interactions, for example the amount of semantic similarity in utterances exchanged between two speakers [26, 27]. Finally, computer-assisted tools are ideally suited to analyse larger bodies of data where manual, conventional coding procedures are not possible or feasible.

### Automated discourse analysis using the Discursis software

The Discursis software [26, 27] is an automated text-analytic tool that allows the investigator to visualise and quantify communication behaviour as two or more speakers engage in a conversation. Previous research has successfully used Discursis to characterise conversation dynamics associated with successful versus unsuccessful doctor-patient consultations [36] and open disclosure conversations about adverse events at hospitals [28], as well as interaction dynamics in television interviews and telephone calls [27]. Furthermore, in a recently published analysis of the current dataset, it was demonstrated how Discursis could be used to qualitatively visualise conceptual engagement between people with dementia and care staff [37]. This approach facilitated the visual identification of topics within a conversation that led to engagement of the person with dementia and rich interaction between both speakers through sharing the same terms.

Apart from being a conversation visualisation tool, Discursis can be used to provide quantitative measures of multiple aspects of conversation behaviour, expressed as a set of ‘Discursis metrics’ (see [27] for details). The software tool processes a conversational transcript by detecting and quantifying the recurrence of terms or concepts throughout the conversation. For each turn in a conversation, the software counts how often, after being first introduced, a given term or concept is referred back to, either by the speaker who first brought it up (self-recurrence) or by their conversation partner (other-recurrence). Furthermore, Discursis counts how much the content of each turn in a conversation recurs in forthcoming turns. Once a conversation has been processed in Discursis, metrics of interest can be exported from the software for subsequent statistical analysis.

### Study Aims

While the communication strategies included in the MESSAGE programme are firmly grounded in theory and informed by empirical research into communication needs in dementia [21], the current knowledge does not extend into an understanding of content-based conversational engagement associated with the use of each recommended strategy. Qualitative and traditional human-coded conversational analysis techniques tend to make it difficult to objectively assess conversational dynamics across large datasets and identify strategies that support conversations. Therefore, the present study’s aims are (i) to empirically identify communication behaviours that, if used by carers, elicit content-based conversational engagement in people with dementia; and (ii) to contribute to the current understanding of the link between computer-assisted output and human coding procedures in the evaluation of content-based conversational engagement between two speakers. Two different series of analyses were conducted to address these aims. Both series of analyses relied on Discursis to efficiently identify instances of content-based conversational engagement in the dataset under investigation.

Analysis 1 aimed to answer the following research question: Is there a link between carer communication behaviour and subsequent content-based engagement of the person with dementia in the conversation? For this analysis, it was hypothesised that spontaneous carer use of specific communication behaviours, as outlined in the MESSAGE training programme, would result in an increased likelihood of content-based engagement by the person with dementia throughout subsequent turns.

Analysis 2 aimed to answer the following research question: Does the computer-generated output of instances of content-based engagement by the care staff reflect human-generated codings of specific facilitative behaviours? It was hypothesised that some communication behaviours would show alignment with Discursis-indicated content-repetition. For example, care staff member behaviour coded as ‘Active listening’, which is defined as care staff restating content provided by the person with dementia, was hypothesised to align with Discursis metrics of other-repetition. It was furthermore hypothesised that a subset of communication behaviours might entail dynamics that the Discursis metrics used in the current analysis cannot express (e.g. ‘Humour’ where an utterance contains humorous content).

## Materials and Methods

### The dataset

Twenty conversations between people with dementia (PWD) living in residential aged care facilities and care staff members (CS) were analysed for the purposes of the current study. The data were recorded as part of a larger research project investigating carer use of strategies for memory and communication support in dementia [24]. Conversations reported here constitute baseline recordings made before any carer training on the MESSAGE strategies had occurred, that is, the current dataset reflects spontaneous communication behaviour by the CS in their conversations with PWD. All procedures were approved by The University of Queensland Human Research Ethics Committee, the service providers’ Human Research Ethics Committees and by the representatives of participating residential aged care facilities. All investigations were conducted according to the principles expressed in the Declaration of Helsinki.

#### Participants

Participants in the conversations were 13 women and 7 men with dementia, along with 14 CS. Prior to research participation, written consent was provided by all CS and PWD and assent was sought from each CS and PWD on the day that the conversations were being recorded. For those PWDs who could not legally provide informed consent on their own behalf and who assented to participating after learning about the study, a legally authorised person provided written consent on the PWD’s behalf.

Inclusion criteria for PWD were a diagnosis of dementia listed in their chart and being a resident at one of the residential aged care facilities involved in the research. Eligible participants with dementia were recruited via information letters sent to the resident’s family by the managers of the four not-for-profit residential aged care facilities in Queensland, Australia, that supported this research. The majority [15] of PWD who participated had previously been diagnosed as Dementia Not Otherwise Specified, where no specific underlying pathology or disease process was identified, four PWD had a diagnosis of vascular dementia, and one PWD had been diagnosed with Alzheimer’s Disease.

Participating people with dementia were aged 72 to 94 years (mean = 87.4, SD = 4.8). Scores obtained on the Mini-Mental State Examination [38] on the day of testing ranged from 3 to 27, indicating severe cognitive impairment in ten, moderate impairment in nine and mild impairment in one participant [39]. No participants were excluded due to scores obtained on the MMSE or other screening tests.

Care staff members had to meet the inclusion criteria that they worked and had direct contact with PWD as part of their role at the residential aged care facility. Those who met the inclusion criteria were invited to participate in the study by their managers or via flyers. In the larger project, 68 care staff members were involved, but only 20 people with dementia could be recruited to complete the baseline conversations.

The CS were three men and 11 women and were either personal care or nursing assistants (12), nursing staff (1) or diversional therapy staff (1). Of the seven CS who disclosed their age, six were aged between 45 and 59 years and one CS was in his early twenties. Ten of the CS participated in only one conversation, two of the CS were recorded having two separate conversations with two different PWDs, and another two CS participated in three separate conversations each, giving a total of 20 conversational dyads. Dyads were formed pragmatically, based on which of the recruited carers at a given residential aged care facility were available for a conversation at a time that suited the recruited residents with dementia. In each dyad, the conversation partners knew each other beforehand.

#### Person with dementia—care staff conversation recordings

Each dyad was recorded individually. During the conversation, the PWD and CS were seated in a comfortable familiar setting, either in the resident’s room at the aged care facility or in a shared lounge area. Prior to the conversation, a research assistant (RA) asked the CS member and the PWD to have a conversation about a topic or topics of their choice for approximately ten minutes. Conversations were recorded using an Olympus DS-30 digital voice recorder placed in close vicinity of the speakers. The recordings varied in length from 5:04min to 13:01min (mean = 9:04min, SD = 2:19min) depending on the PWD’s willingness to continue the conversation.

On several occasions, the dyads were interrupted by other staff or residents that were not involved in the research, and had not noticed that the PWD and CS were taking part in a research recording. These typically short interruptions were marked in the transcripts and excluded from analysis. All recordings were transcribed into written text using a modified Jeffersonian transcription [40], and entered into SALT (Systematic Analysis of Language Transcripts) Research Version [41] for initial analysis of communication behaviour use by CS. The notation relevant to interpretation of the text and the preparations of transcripts for input to SALT were identical to the procedures described in a previous publication on this dataset by Baker and colleagues [37].

### Labelling of individual utterances

#### Communication behaviour labelling by communication experts

The final SALT conversation transcripts were analysed by authors ERC and RB for a range of communicative behaviours consistent with MESSAGE strategy use. Each utterance was manually labelled for spontaneous use of these communication behaviours by the CS by inserting a behaviour label in brackets. Because no video- or meta-data was recorded alongside the conversations to record non-verbal parameters, behaviours pertaining to non-verbal acts were not coded here (e.g. reducing distractions to maximise attention, and body language and expression). In addition to marking facilitative communication behaviour use, the two communication experts also marked non-facilitative communication behaviours that represented a lack of communication support when required or specifically non-facilitative behaviours, such as interrupting. Table 1 provides examples and definitions of the 22 facilitative and 13 non-facilitative communication behaviours coded in the dataset, together with details on the frequency of their spontaneous use collated for all CS participants.

**Table 1:** Examples, definitions and frequency of use of the communication behaviours coded in the dataset.

Communication behaviour	Label	Definition	Example of text and coding from the dataset	Frequency of communication behaviour
Facilitative:				
**Verbal acknowledgment**	VblAck	Verbally acknowledge/affirm PWD utterance (e.g. mmm, yeah, yeah?, did you?, oh, ah, right)	CS: *What did you play*?	543 (31.2%)
			PWD: *Rugby league*.	
			CS: *Oh*, *did you* **[VblAck]**?	
**Active listening**	ActListen	Show active listening by restating, paraphrasing, developing extending, elaborating on or incorporating PWD content/taking account of what PWD has just expressed	PWD: *Oh*, *of course*, *yes*. *They’re like big kids (laugh)*.	325 (18.6%)
			CS: *They’re like kids* (laugh). *Like big kids* **[ActListen].**	
			PWD: *Just like grown-up kids*, *you know*.	
**Give time**	GiveTime	Give PWD time to respond or continue (shown by a pause left before PWD response/continuation) even if response is TIB or unsuccessful	CS: *Oh*, *just you and your hubby*, *running the farm*.	204 (11.7%)
			PWD: *Mmm (affirm)*.	
			CS: *That must have been a bit hard (SPause)* **[GiveTime].**	
			PWD: *Oh*, *well*, *it’s- it’s hard work*, *naturally*.	
**PWD knowledge**	PWDKnowl	Show evidence for incorporating prior knowledge (gained before this conversation) of PWD’s life and interests	CS: *And you’ve got brothers and sisters*, *haven’t you* **[PWDKnowl]**?	83 (4.8%)
			PWD: *I had brothers*. *Two of them died*.	
**Verbal encouragement to continue**	VblEncCont	Verbal encouragers with intonation simply to continue (mmm), not to acknowledge or affirm responses	PWD: *I had some bait there*.	72 (4.1%)
			CS: *Mmm* **[VblEncCont]**.	
			PWD: *I put a bait on*, *and I had a little bit of a pick*.	
			CS: *Mmm* **[VblEncCont]**.	
			PWD: *I missed him*.	
**Expand**	Expand	CS gives additional explanation, clues or clarification to support PWD comprehension	CS: *Did you have to make cocktails […]*?	69 (4.0%)
			PWD: *What do you mean*?	
			CS: *You know*, *those fancy drinks* **[Expand]**.	
**Answer content**	AnsCont	CS provides/suggests answer content, choice/alternatives or examples in the question.	CS: *And did you have any animals*, *such as chickens*, *goats and sheep* **[AnsCont]**?	57 (3.3%)
			PWD: *No*, *we had mostly cows*.	
**Use name**	UseName	Address PWD by name	CS: *How you going*, *Fred* ^*a*^ **[UseName]**?	56 (3.2%)
**Self disclosure**	SelfDisc	Self disclosure by CS, e.g. sharing personal circumstances and experiences	CS: *My favourite colour is green* **[SelfDisc]**. *What colours do you like*?	54 (3.1%)
**Rephrase**	Rephrase	CS rephrases their own utterance when there is TIB using different words or a markedly different syntactic structure	CS: *Did you [sen-] give them their marching orders*?	48 (2.8%)
			PWD: *Hmm*?	
			CS: *Did you tell them you have to go home now*, *you’ve had enough* **[Rephrase]**?	
**Work out**	WorkOut	CS tries to work out PWD’s message	CS: *What work did you do […]*?	38 (2.2%)
			PWD: *Radio (unintelligible)*.	
			CS: *Were you a radio announcer* **[WorkOut]**?	
**Humour**	Humour	CS utterance contains humorous content	CS: *What’s on TV this afternoon*?	36 (2.1%)
			PWD: *I wouldn’t know*, *wouldn’t have a clue*.	
			CS: *‘Days of our lives’* (SPause)?	
			PWD: *Is it*?	
			CS: *Yeah*, *‘Young and the useless’* **[Humour]**.	
			PWD: *(laughs)*	
**PWD topic**	PWDTopic	CS takes up PWD-initiated topic	PWD: *I made sure she had a good education*, *‘cause her old man hasn’t*. *[…]*	36 (2.1%)
			CS: *Yeah*, *you’ve got to have an education these days* **[PWDTopic]**.	
**Reduce question**	ReduceQ	Yes-No alternative questions are provided when an open question fails or elicits non-specific answer or when a more specific answer was expected	CS: *[…] When did you come to Australia*?	36 (2.1%)
			PWD: *Ah (SPause}*. *Mmm (SPause)*.	
			CS: *Was it after the war* **[ReduceQ]**?	
**Visual topic**	VisTopic	Utterances accompanying gesture/pointing for a topic in view, a visual cue in setting (e.g. photos)	CS: *Who is this*, *in the photo* **[VisTopic]**?	35 (2.0%)
			PWD: *Me*.	
**Rephrase question**	RephQ	Immediate rephrasing of a question without changing information required (to clarify or better specify)	CS: *Where did that saying come from*? *Who used to say that* **[RephQ]**?	29 (1.7%)
**Suggest content**	SugCont	Suggest content to help with word finding or to complete PWD utterance	CS: *And what’s your favourite flower*?	21 (1.2%)
			PWD: *Um*, *favourite was black and white one*. *I forget now how you call them* (SPause).	
			CS: *Hmm*, *carnations* **[SugCont]**?	
			PWD: *Yeah*.	
**Mixed question support**	MixedQ	Yes-No or alternatives question immediately after open question, regarding the same information (no pause between questions)	CS: *And what hobbies did you have*, *did you have any other hobbies* **[MixedQSupport]**?	14 (0.8%)
			PWD: *Just fishing*.	
**Managing distress**	ManDis	Manage distress or potentially upsetting topics sensitively	PWD: *My memory is very weak*.	13 (0.7%)
			CS: *But you remember some things*, *you [tol-] you’ve taught me lots* **[ManDis]**.	
**Managing confusion**	ManConf	Manage confusion or memory problem sensitively without arguing; CS reassures rather than contradicts	PWD: *I couldn’t remember at all*, *it’s just one of those things*, *you know […]*.	8 (0.5%)
			CS: *But that happens when we get older*, *doesn’t it* **[ManConf]**?	
**Attention orientation**	AttOrient	Orient for attention (explicitly)	CS: *What about gardening (XLPause)*? *What about gardening (LPause)*? *Margaret* ^*a*^ *(MPause)* **[AttOrient]**?	3 (0.2%)
**Reduce choice**	ReduceChoice	Reduction of an alternatives question to a single Yes-No choice question	CS: *Who*?	1 (0.1%)
			PWD: *Samantha* ^a^.	
			CS: *What*, *one of the workers*, *or one of the- or the owner of the pub*?	
			PWD: *Yeah*.	
			CS: *Was she the owner* **[ReduceChoice]**?	
**Non-facilitative:**				
**No time**	NoTime	Insufficient time left for PWD to respond or complete response	PWD: Those memories and that come to me and *wake-*	78 (4.5%)
			CS: (interruption) **[NoTime]** *What sort of memories*?	
**No active listen**	NoActListen	PWD meaning is ignored, disregarded or overridden	CS: *Who was best*, *you or her*?	48 (2.8%)
			PWD: *[I-I-I] I think I was the strongest*.	
			CS: *She was strong* **[NoActListen]**. *Did she win any trophies*?	
**No managing confusion**	NoManConf	CS argues/contradicts rather than reassures PWD; or CS reiterates/echoes a memory problem (e.g., I can’t remember/I don’t know) mentioned by PWD	PWD: *My memory- my memory- memory is terrible (SPause)*.	25 (1.4%)
			CS: *Your memory’s terrible* **[NoManConf]**.	
**No clear referent**	NoClearRef	Use of expression (e.g., pronoun, deictic word) where referent is not clear	CS: *Did you drive there*, *or did you live up that way* **[NoClearRef]**?	20 (1.1%)
			PWD: *Hmm*?	
			CS: *Did you used to drive to work*, *or did you live that near*?	
**Multiple questions**	MultQ	Questions asking for different information with no time in-between to answer	CS: *Or you like tennis*? *Or you like golf* **[MultQ]**?	18 (1.0%)
**No repetition**	NoRep	No repeat or rephrase to assist after TIB	CS: *Hmm*. *And did you get baked beans this morning or spaghetti (MPause)*?	11 (0.6%)
			PWD: *Yeah*.	
			CS: **[NoRep]** *Cause we’re g- we’re getting the hot breakfast again on a Thursday*.	
**No clear topic**	NoClearTopic	Topic not made clear when introduced, refreshed or changed to help PWD keep track	CS: *Yeah*, *that’s really good*.	11 (0.6%)
			PWD: *Mmm (affirm)*	
			CS: *What about cooking (SPause)* **[NoClearTopic]**?	
**Remember**	RemQ	Follow-up questions: Do you/can you remember/recall/know…?	CS: *And [wh-] where did you live before*?	10 (0.6%)
**question**			PWD: *Ah*.	
			CS: *Can you remember* **[RemQ]**?	
**No work out**	NoWorkOut	CS is not trying to work out what PWD is trying to say	CS: *Oh*, *sausage maker*, *oh*. *Did you ever win the best sausage maker*? *At the butchers*?	7 (0.4%)
			PWD: *No*. *Um*, *we had (SPause) factory*.	
			CS: *Yeah* **[NoWorkOut**].	
			PWD: *Yeah*.	
**Self repetition**	SelfRep	CS repeats own utterance with neither a pause nor a TIB in between.	CS: *What’s your daughter-in-law name*? *What*, *what her name* **[SelfRep]**?	5 (0.3%)
**Test question**	TestQ	Asking for information that is already known for purposes of probing memory abilities	CS: *Do you know where you live* **[TestQ]**?	4 (0.2%)
			PWD: *Here*.	
			CS: *Yeah*, *do you know what this is called* **[TestQ]**?	
			PWD: *Oh golly*, *never thought about it*.	
**Talk down**	TalkDown	Speaking in a condescending manner (e.g. babytalk or ‘elderspeak’)	PWD: *Oh well*, *she’ll- she’ll come back*.	2 (0.1%)
			CS: *Yeah*. *Does she love her mother* **[TalkDown]**?	
**No familiar word**	NoFamWord	Use of low frequency/difficult word	CS: *Nuh [VblAck]*. *What do you reckon you suit best*, *you look good in (SPause)* **[NoFamWord]**?	1 (0.1%)
			PWD: *Suit*?	
			CS: *Suit*. *Yeah*, *what- what colour did the suit- the suits you the best*?	

^a^Name changed from original data for de-identification purposes

Abbreviations: CS–care staff, PWD–person with dementia, TIB–trouble-indicating behaviour, SPause–short pause, MPause–medium-length pause

#### Quantifying conversational engagement using Discursis

For each of the 20 conversations, the SALT-prepared and communication behaviour labelled conversation transcript was exported from the SALT format into a comma-separated file that contained each line of text as a row in a column, assigned to the respective speaker who made the utterance (CS or PWD). This conversation data was subsequently loaded into Discursis, creating a separate project for each conversation, to assess the unique recurrence of content between two speakers within a given conversation. In line with previously optimised Discursis processing parameters for this dataset [37], term-based recurrence for a maximum of 200 terms was chosen, and a list of stopwords was fed into the Discursis processor to ensure semantically empty terms such as ‘no’, ‘hmm’ and ‘yeah’, as well as potentially ambiguous terms such as ‘right’ and ‘like’ were not included in the computation of shared recurrence between speakers. Additionally, the Discursis option to automatically detect meta data, such as the coded communication behaviour labels provided in brackets within each turn, was enabled, which meant that for each utterance line, the Discursis output file would attach a label if a particular coded behaviour occurred in a given utterance.

Following these initial steps, the transcripts for each conversation were processed in Discursis, resulting in 20 datasets that detailed for each conversation and each utterance who spoke (CS or PWD), what was being said (text), results for six different Discursis metrics of interest (see below) as well as Discursis-provided metadata labels of coded communication behaviour per utterance. These highly detailed individual conversation datasets were subsequently collated to form a single dataset containing utterance-by-utterance data on all variables of interest. The final dataset had a total of 3,460 observations (single utterances) available for analysis.

There are twelve basic variables that can be exported as part of the Discursis quantitative analysis, termed the Discursis primitive metrics (primitives). Each primitive expresses recurrence occurring along a unique combination of three recurrence dimensions: time scale (short-, medium-, long-term), direction (forward, backward) and type (self, other). Short-term metrics express conversation behaviour between two consecutive turns, either in relation to one’s own turn (‘self backward short’ and ‘self forward short’) or the other speaker’s immediately preceding or following turn (‘other backward short’ and ‘other forward short’ respectively). Medium-range metrics are calculated on the basis of ten turns in either direction, and long-range metrics are calculated across all turns within a conversation. Depending on the research question of interest, various Discursis primitives can be used individually for analysis, but it is also possible to combine the Discursis primitive metrics as building blocks for the computation of novel variables that reflect a given conversational behaviour under investigation [27].

The conversational behaviour of interest in the current investigation is the sharing of content between two speakers, which is quantified in metrics pertaining to ‘other’-recurrence. To account for content-based conversational engagement across multiple time dimensions, the current investigation will focus on ‘other’ recurrence in the short-, medium- and long-term across the conversation. Given that the communication behaviours of interest (MESSAGE strategy related behaviours) relate specifically to CS communication, corresponding labels were present only on CS turns, and therefore it was the CS turns that were of particular interest for the current turn-wise investigation.

From the perspective of the CS turns, the ‘other backward—short, medium and long’ metrics indicate whether or not the CS is engaging with PWD content across the different timescales. The ‘other forward—short, medium and long’ metrics from the CS perspective indicate whether or not PWD is engaging with CS content. Table 2 provides an example of how CS-other-related metrics correspond to term-recurrence in consecutive utterances in a CS-PWD conversation, alongside corresponding binary labels (Yes or No) indicating for each CS turn whether other-related recurrence was present or not in the short-, medium- and long-range.

**Table 2:** Examples of binary labels assigned to each utterance indicating the presence or absence of shared recurrence between speaker.

		Other backward Discursis metrics^a^			Other forward Discursis metrics^b^		
Channel	Text	Short	Medium	Long	Short	Medium	Long
PWD	*No*, *they were pretty good*.	0	0	0	0	0	0
CS	*Were they* [VblAck]?	0 (No)^c^	0 (No)	0 (No)	0 (No)	0 (No)	0 (No)
PWD	*Yeah they- you- you could pet them and they wouldn't* ***yelp*.**	0	0	0	0.354	0.071	0.009
CS	*Oh yeah they're horrors aren't they when they* ***yelp*** [ActListen].	0.354 (Yes)^d^	0.071 (Yes*)	0.004 (Yes*)	0 (No)	0 (No)	0 (No)
PWD	*No*, *god that's right*, *it drives you k- crazy*.	0	0	0	0	0	0
CS	*It's always the little ones isn't it that do that*?	0 (No)	0 (No)	0 (No)	0 (No)	0 (No)	0 (No)
PWD	*Yes*.	0	0	0	0	0	0
CS	*The big ones don't seem to* ***bark*** *a lot* [Expand].	0 (No)	0 (No)	0 (No)	0.707 (Yes)	0.177 (Yes*)	0.021 (Yes*)
PWD	*No they don't* ***bark*** *so much*.	0.707	0.141	0.007	0	0.250	0.029
CS	*No* [VblAck].	0 (No)	0 (No)	0 (No)	0 (No)	0 (No)	0 (No)
PWD	*No*, *I- I found that out*.	0	0	0	0	0	0
CS	*I've got- I've got a labrador at home* [SelfDisc].	0 (No)	0 (No)	0 (No)	0 (No)	0 (No)	0 (No)
PWD	*Oh yes*.	0	0	0	0	0	0
CS	*And she doesn't* ***bark***.	0 (No)	0.200 (Yes)	0.010 (Yes*)	0 (No)	0 (No)	0 (No)

^a^ *Other backward Discursis metrics* quantify the extent to which a given utterance shares content from previous utterances made by the conversation partner. *Other backward short* indicates sharing of content from the immediately preceding utterance; *other backward medium* indicates sharing of content that a conversation partner uttered within a range of 10 preceding turns; *other backward long* indicates sharing of content that a conversation partner uttered across all preceding utterances.

^b^ *Other forward Discursis metrics* quantify the extent to which content of a given turn is being shared forward by the conversation partner in subsequent turns. *Other forward short* indicates that the conversation partner is sharing content of a given utterance in the immediately following utterance; *other forward medium* indicates that content of a given turn is being shared in one or several subsequent turns by the conversation partner within a range of ten utterances; *other forward long* indicates forward sharing of any content occurring across all subsequent turns.

^c, d^ Binary labels (Yes or No) for each care staff (CS) utterance indicate whether CS is sharing terms previously used by the person with dementia (PWD) and whether PWD is sharing terms previously used by CS.

Yes*—despite Discursis values indicating medium- and long-term sharing for these turns, these values can be ascribed to the presence of short-term engagement and were therefore not included as instances of medium- and long-term recurrence in the analysis.

Abbreviations: CS—Care staff, PWD–Person with dementia, VblAck–Verbal acknowledgment (coded communication behaviour), ActListen–Active listening (coded communication behaviour), SelfDisc–Self disclosure (coded communication behaviour), Y–Yes, N—No

Using these metrics from the perspective of the CS, the Discursis-based outcomes relating to conversational engagement and the presence or absence of coded communication behaviour by CS could be analysed in relation to one another within each turn. It is important to note that by default, Discursis counts instances of short-term recurrence into the computation of medium- and long-term recurrence and instances of medium-term recurrence into long-term recurrence. Therefore, medium-term recurrence was only coded for if it occurred in isolation from short-term recurrence and long-term recurrence was only coded for if it occurred in isolation from short- and medium-term recurrence (see Table 2).

### Statistical analysis

#### Analysis 1: Which communication behaviours elicit PWD engagement?

In order to assess whether communication behaviours that are representative of MESSAGE strategies co-occurred with Discursis-indicated PWD engagement with CS content, a series of logistic regression analyses was performed. In separate analyses, each coded communication behaviour was entered as a predictor variable of short-, medium- and long-range PWD engagement, performing separate analyses for each range. The dependent variables of interest in the statistical analyses performed for analysis 1 were three binary variables indicating whether or not PWD was engaging with CS content (CS-other forward short, CS-other forward medium, and CS-other forward long; see Table 2).

#### Analysis 2: Alignment of human and computer-generated labelling

In order to assess whether there was overlap between communication behaviours and CS engagement with previous PWD content, a second series of logistic regression analyses was performed. In separate analyses, each of the facilitative or non-facilitative communication behaviours were entered as a predictor variable of short-, medium- and long-range CS engagement, performing separate analyses for each range. The dependent variables of interest in the statistical analyses performed for analysis 2 were three binary variables indicating whether or not CS was engaging with PWD content (CS-other backward short, CS-other backward medium, and CS-other backward long; see Table 2). Both in analysis 1 and analysis 2 only communication behaviours that occurred in 1% (18 turns) or more of all CS turns were included (see Table 1 for frequency of communication behaviour use).

## Results

### Analysis 1: Which communication behaviours elicit PWD engagement?

Out of the total of 22 facilitative and non-facilitative communication behaviours that were included in the analysis, nine of the facilitative communication behaviours were found to significantly increase the likelihood of PWD content-based engagement in the short-, medium-, and/or long-term, while one facilitative behaviour was found to significantly decrease the likelihood of PWD engagement. None of the five non-facilitative communication behaviours included in the analysis had a significant impact on PWD engagement. Table 3 provides results obtained for analysis 1.

**Table 3:** Results of logistic regression analyses for CS other forward.

CS Communication behaviour (CB) ^a^	PWD engagement with CS content (CS forward other)	*p*	OR (95% CI)	Probability of PWD engagement when CB was present	Odds of PWD engagement when CB was present	Odds of PWD engagement when CB was not present
***Facilitative***						
**Verbal**	Short	.064	.718 (.506–1.020)	8%	46/497	137/1063
**acknowledgment**	Medium*** ^(-)^	< .001	.342 (.196–.596)	3%	15/528	92/1108
	Long*** ^(-)^	.004	.556 (.374–.828)	6%	33/510	125/1075
**Active listening**	Short*** ^(+)^	.002	1.757 (1.238–2.493)	15%	50/275	133/1285
	Medium* ^(+)^	.022	1.683 (1.079–2.625)	9%	29/296	78/1340
	Long*** ^(+)^	< .001	1.991 (1.382–2.867)	14%	47/278	111/1307
**Give time**	Short*** ^(+)^	.001	1.946 (1.303–2.908)	17%	35/169	148/1391
	Medium	.647	1.146 (.640–2.050)	7%	14/190	93/1446
	Long	.898	.967 (.578–1.617)	9%	18/186	140/1399
**PWD knowledge**	Short** ^(+)^	.009	2.135 (1.209–3.769)	19%	16/67	167/1493
	Medium*** ^(+)^	< .001	3.419 (1.855–6.301)	17%	14/69	93/1567
	Long	.083	1.753 (.929–3.307)	14%	12/71	146/1514
**Verbal**	Short	.086	.360 (.112–1.156)	4%	3/69	180/1491
**encouragement to continue**	Medium	.121	.208 (.029–1.511)	1%	1/71	106/1565
	Long	.076	.277 (.067–1.142)	3%	2/70	156/1515
**Expand**	Short	.272	1.471 (.739–2.927)	14%	10/59	173/1501
	Medium	.370	1.483 (.627–3.509)	8%	6/63	101/1573
	Long	.457	1.331 (.626–2.837)	12%	8/61	150/1524
**Answer content**	Short*** ^(+)^	< .001	3.892 (2.158–7.018)	30%	17/40	166/1520
	Medium	.779	1.160 (.412–3.267)	7%	4/53	103/1583
	Long	.585	.751 (.268–2.102)	7%	4/53	154/1532
**Use name**	Short	.171	1.665 (.802–3.456)	16%	9/47	174/1513
	Medium*** ^(+)^	.003	3.105 (1.479–6.519)	16%	9/47	98/1589
	Long	.663	1.212 (.511–2.873)	11%	6/50	152/1533
**Self disclosure**	Short	.882	1.068 (.451–2.531)	11%	6/48	177/1512
	Medium* ^(+)^	.039	2.367 (1.043–5.370)	13%	7/47	100/1589
	Long*** ^(+)^	.001	3.020 (1.555–5.863)	22%	12/42	146/1543
**Rephrase**	Short	.647	1.225 (.514–2.923)	13%	6/42	177/1518
	Medium	.567	.658 (.158–2.750)	4%	2/46	105/1590
	Long	.858	.910 (.323–2.566)	8%	4/44	154/1541
**Work out**	Short	.114	1.962 (.851–4.521)	18%	7/31	176/1529
	Medium	.378	.408 (.055–3.000)	3%	1/37	106/1599
	Long	.800	.857 (.261–2.819)	8%	3/35	155/1550
**Humour**	Short	.160	.239 (.033–1.758)	3%	1/35	182/1525
	Medium	.581	1.401 (.423–4.645)	8%	3/33	104/1603
	Long* ^(+)^	.034	2.487 (1.072–5.774)	19%	7/29	151/1556
**PWD topic**	Short	.338	.496 (.118–2.081)	1%	2/34	181/1526
	Medium	.059	2.538 (.966–6.665)	14%	5/31	102/1605
	Long* ^(+)^	.034	2.487 (1.072–5.774)	19%	7/29	151/1556
**Reduce question**	Short	.504	1.385 (.532–3.608)	14%	5/31	178/1529
	Medium	.883	.897 (.213–3.787)	6%	2/34	105/1602
	Long	.313	1.638 (.628–4.274)	14%	5/31	153/1554
**Visual topic**	Short	.998	.000	0%	0/35	183/1525
	Medium	.547	1.446 (.436–4.800)	9%	3/32	104/1604
	Long	.624	1.302 (.454–3.737)	11%	4/31	154/1554
**Rephrase question**	Short*** ^(+)^	.004	3.350 (1.462–7.677)	28%	8/21	175/1539
	Medium	.864	1.135 (.266–4.838)	7%	2/27	105/1609
	Long	.683	.740 (.174–3.140)	7%	2/27	156/1558
**Suggest content**	Short	.571	1.428 (.417–4.894)	14%	3/18	180/1542
	Medium	.998	.000 (.000–.000)	0%	0/21	107/1615
	Long	.498	.498 (.066–3.739)	5%	1/21	157/1565
***Non-facilitative***						
**No time**	Short	.759	1.118 (.548–2.278)	12%	9/69	174/1491
	Medium	.560	1.290 (.548–3.040)	8%	6/72	101/1564
	Long	.708	1.154 (.545–2.445)	10%	8/70	150/1515
**No active listening**	Short	.064	2.017 (.961–4.235)	19%	9/39	174/1521
	Medium	.567	.658 (.158–4.729)	4%	2/46	105/1590
	Long	.494	.662 (.203–2.156)	6%	3/45	155/1540
**No managing confusion**	Short	.683	.738 (.173–3.158)	8%	2/23	181/1537
	Medium	.656	.634 (.085–4.729)	4%	3/22	287/1431
	Long	.608	1.375 (.407–4.646)	12%	6/19	442/1276
**No clear referent**	Short	.173	2.156 (.713–6.521)	20%	4/16	179/1544
	Medium	.475	1.712 (.392–7.478)	10%	2/18	105/1618
	Long	.531	.525 (.070–3.948)	5%	1/19	157/1566
**Multiple questions**	Short	.115	2.468 (.804–7.578)	22%	4/14	179/1546
	Medium	.385	1.929 (.438–8.499)	11%	6/12	284/1441
	Long	.606	.587 (.078–4.444)	6%	7/11	441/1284

^a^ Facilitative and non-facilitative communication behaviours are listed in descending order according to their frequency of use across all conversations

Abbreviations: CS–Care staff, CB–Communication behaviour, PWD–Person with dementia, OR–Odds ratio, CI–Confidence interval

* significant, p < .05

** significant, p < .01

*** significant, p < .005

^(+)^ if the communication behaviour was used, the odds of content-based engagement were significantly increased

^(-)^ if the communication behaviour was used, the odds of content-based engagement were significantly decreased

#### Communication behaviours associated with computer-identified PWD engagement

Relying on the *p*-value, odds ratio and probability combined for interpretation of the results (see Table 2), the use of ‘Answer content’ was found to be the strongest predictor of subsequent PWD engagement, with a 30% probability of subsequent short-term PWD engagement if the behaviour occurred (*p* < .001, Odds Ratio [OR] = 3.892). Other facilitative communication behaviours that reliably elicited PWD engagement in the short term were ‘Rephrase question’ (28%, *p* = .004, OR = 3.350), ‘PWD knowledge’ (19%, *p* = .009, OR = 2.135), ‘Give time’ (17%, *p* = .001, OR = 1.946) and ‘Active listening’ (15%, *p* = .002, OR = 1.757).

Facilitative communication behaviours that elicited medium-term PWD engagement were ‘PWD knowledge’ (17%, *p* = < .001, OR = 3.419), ‘Use name’ (16%, *p* = .003, OR = 3.1505), ‘Self disclosure’ (13%, *p* = .039, OR = 2.367) and ‘Active listening’ (9%, *p* = .022, OR = 1.683), while long-term PWD engagement was predicted by ‘Self disclosure’ (22%, *p* = .001, OR = 3.020), ‘PWD topic’ (19%, *p* = .034, OR = 2.487), ‘Humour’ (19%, *p* = .034, OR = 2.487) and ‘Active listening’ (14%, *p* = .001, OR = 1.991).

**Fig 1:**
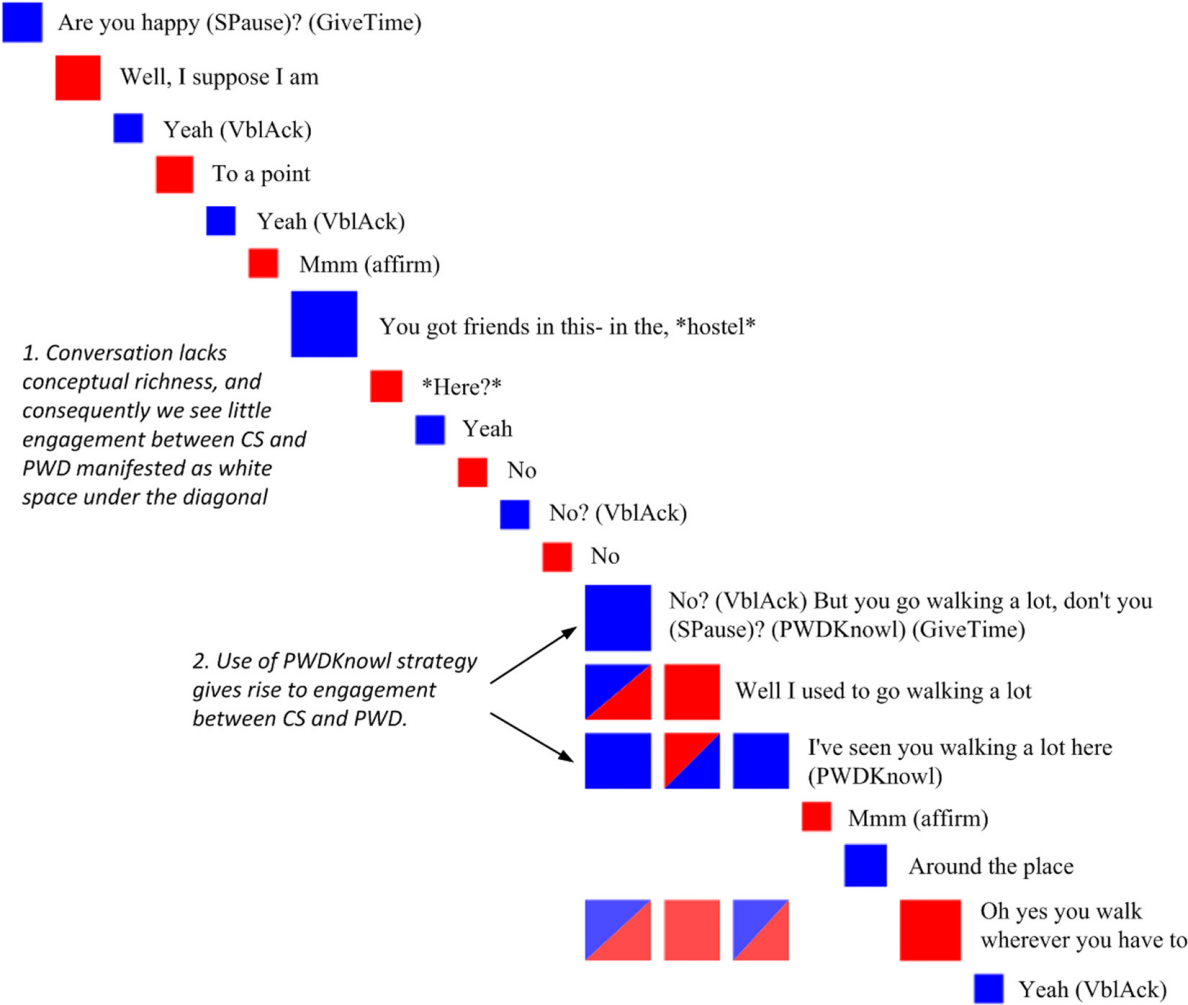
Zoom-in view of a Discursis plot shows how use of the coded communication behaviour ‘PWD knowledge’ (PWDKnowl), aligns with forward recurrence where the person with dementia (PWD) engages with content previously provided by the care staff member (CS). Blue boxes along the diagonal represent care staff member turns. Red boxes along the diagonal represent turns by the person with dementia. Red-blue shaded boxes under the diagonal indicate instances of shared recurrence.

A visualisation of the impact of CS communication behaviour on subsequent PWD engagement can be found in Fig 1, showing term-based engagement before and after the CS used ‘PWD knowledge’, which was found to predict short- and medium-term PWD engagement. Spontaneous use of this behaviour was coded whenever the CS mentioned information relevant to the PWD’s life and interests known to CS prior to the conversation (see Table 1 for communication behaviour definitions). Fig 1 presents a zoom-in view of a Discursis plot of an excerpt from a conversation between CS and PWD. Utterances made by CS are presented as blue boxes and utterances made by PWD are presented in red. The diagonal of blue and red boxes running from the upper left to the lower right corner represents the time course of the conversation and the space under the diagonal indicates whether PWD is engaging with CS content (blue/red shaded boxes under the diagonal) and whether CS is engaging with PWD content (red/blue shaded boxes under the diagonal). Single-coloured boxes under the diagonal indicate self-recurrence.

In the initial twelve utterances shown in Fig 1, the CS and PWD utterances lack conceptual richness and mutual sharing of content. From turn 13 onwards, the dynamics change and content-based engagement is present in relation to the PWD’s habit of going for walks. This content-based engagement was likely elicited because in turn 13 CS used prior knowledge about walking that he or she has about the PWD to discuss a familiar topic, i.e. CS uses the coded behaviour ‘PWD knowledge’.

#### Predictors of a lack of PWD engagement

None of the five non-facilitative communication behaviours under investigation were found to significantly affect the probability of PWD engagement. However, one of the facilitative behaviours (‘Verbal acknowledgment’) was found to be a significant predictor of a lack of content-based PWD engagement in subsequent turns in the medium-range (3%, *p* < .001, OR = .342) and long-term (6%, *p* = .004, OR = .556). ‘Verbal acknowledgment’ was defined as instances in a conversation where CS provided non-lexical (‘mmh’) or phrasal (‘did you?’) backchannelling responses to the PWD.

### Analysis 2: Alignment of human- and computer-generated labelling

Eight facilitative behaviours out of the total of 22 facilitative and non-facilitative communication behaviours included in the analysis were found to reliably co-occur with CS engagement with PWD content. Two additional facilitative behaviours were found to predict an absence of CS engagement with PWD content. Table 4 provides results obtained for analysis 2.

**Table 4:** Results of logistic regression analyses for CS other backward.

CS Communication behaviours (CB) ^a^	CS engagement with PWD content (CS other backward)	*p*	OR (95% CI)	Probability of CS engagement when CB was present	Odds of CS engagement when CB was present	Odds of CS engagement when CB was not present
***Facilitative***						
**Verbal**	Short*** ^(-)^	< .001	.228 (.148–.349)	5%	25/518	210/990
**acknowledgment**	Medium	.510	.858 (.543–.1.354)	5%	27/516	69/1131
	Long	.317	.826 (.568–1.201)	8%	41/502	108/1092
**Active listening**	Short*** ^(+)^	< .001	20.411 (14.743–28.259)	51%	166/159	69/1349
	Medium	.609	.866 (.499–1.502)	5%	16/309	80/1338
	Long* ^(-)^	.011	.500 (.294 –.853)	5%	16/309	133/1285
**Give time**	Short	.327	1.224 (.817–1.836	16%	32/172	203/1336
	Medium** ^(+)^	.005	2.092 (1.249–3.505)	10%	20/184	76/1463
	Long* ^(+)^	.046	1.594 (1.009–2.517)	12%	25/179	124/1415
**PWD knowledge**	Short	.552	1.203 (.655–2.211)	16%	13/70	222/1438
	Medium	.442	.632 (.196–2.039)	4%	3/80	93/1567
	Long	.445	1.320 (.647–2.695)	11%	9/74	140/1520
**Verbal encouragement**	Short	.053	.367 (.133–1.015)	6%	4/68	231/1440
**to continue**	Medium	.612	.738 (.228–2.388)	4%	3/69	93/1578
	Long	.056	.145 (.020–1.051)	1%	1/71	148/1523
**Expand**	Short	.640	.836 (.395–1.770)	12%	8/61	227/1447
	Medium	.511	.802 (.416–1.546)	16%	11/58	320/1354
	Long	.358	1.426 (.669–3.040)	12%	8/61	141/1533
**Answer content**	Short	.155	.475 (.170–1.326)	7%	4/53	231/1455
	Medium** ^(+)^	.001	3.446 (1.638–7.251)	16%	9/48	87/1588
	Long	.588	1.269 (.536–3.009)	11%	6/51	143/1543
**Use name**	Short	.565	1.238 (.598–2.560)	16%	9/47	226/1461
	Medium	.997	.000 (.000 –.000)	0%	0/56	96/1591
	Long	.703	.818 (.292–2.294)	7%	4/52	145/1542
**Self disclosure**	Short	.771	1.120 (.522–2.404)	15%	8/46	227/1462
	Medium	.074	2.221 (.926–5.327)	11%	6/48	90/1599
	Long*** ^(+)^	< .001	3.621 (1.894–6.922)	24%	13/41	136/1553
**Rephrase**	Short	.149	.420 (.130–1.364)	6%	3/45	232/1463
	Medium* ^(+)^	.038	2.548 (1.055–6.150)	13%	6/42	90/1605
	Long* ^(+)^	.013	2.563 (1.217–5.400)	19%	9/39	140/1555
**Work out**	Short	.172	1.736 (.786–3.835)	21%	8/30	227/1478
	Medium*** ^(+)^	< .001	4.900 (2.182–11.002)	21%	8/30	88/1617
	Long	.884	.915 (.278–3.012)	8%	3/35	146/1559
**Humour**	Short	.367	.578 (.176–1.900)	8%	3/33	232/1475
	Medium	.990	1.009 (.239–4.265)	6%	2/34	94/1613
	Long	.963	.972 (.295–3.208)	8%	3/33	146/1561
**PWD topic**	Short	.294	1.566 (.678–3.616)	19%	7/29	228/1479
	Medium*** ^(+)^	< .001	11.059 (5.410–22.606)	36%	13/23	83/1624
	Long*** ^(+)^	< .001	5.003 (2.410–10.383)	31%	11/25	138/1569
**Reduce question**	Short	.942	1.036 (.399–2.691)	14%	5/31	230/1477
	Medium	.478	.485 (.066–3.577)	3%	1/35	95/1612
	Long	.963	.972 (.295–3.208)	8%	3/33	146/1561
**Visual topic**	Short* ^(+)^	.037	2.270 (1.050–4.907)	26%	9/26	226/1482
	Medium	.496	.499 (.068–3.687)	3%	1/34	95/1613
	Long	.548	.644 (.153–2.709)	6%	2/33	147/1561
**Rephrase question**	Short	.306	.471 (.111–1.993)	7%	2/27	233/1481
	Medium	.742	.1.277 (.299–5.449)	7%	2/27	94/1620
	Long	.100	2.271 (.854–6.043)	17%	5/24	144/1570
**Suggest content**	Short	.914	1.070 (.313–3.662)	17%	3/18	232/1490
	Medium	.998	.000 (.000–.000)	0%	0/21	96/1626
	Long	.998	.000 (.000–.000)	0%	0/21	477/1245
***Non-facilitative***						
**No time**	Short	.395	.724 (.344–1.525)	11%	8/70	227/1438
	Medium	.881	.924 (.331–2.583)	5%	4/74	92/1573
	Long	.336	1.421 (.694–2.907)	8%	6/69	140/1525
**No active listening**	Short	.530	.741 (.290–1.889)	10%	5/43	230/1465
	Medium	.139	2.050 (.793–5.299)	10%	5/43	91/1604
	Long	.565	.707 (.217–2.304)	6%	3/45	146/1549
**No managing confusion**	Short	.341	1.617 (.601–4.532)	20%	5/20	230/1488
	Medium	.585	1.502 (.349–6.468)	8%	2/23	94/1624
	Long	.537	1.468 (.434–4.964)	12%	3/22	146/1572
**No clear referent**	Short	.648	.711 (.164–3.082)	10%	2/18	233/1490
	Medium	.384	1.926 (.440–8.422)	10%	2/18	94/1629
	Long	.816	1.191 (.274–5.184)	10%	2/18	147/1576
**Multiple questions**	Short	.692	1.287 (.370–4.480)	17%	3/15	232/1493
	Medium	.050	3.510 (.998–12.337)	17%	3/15	93/1632
	Long	.697	1.342 (.306–5.893)	11%	2/16	147/1578

^a^ Facilitative and non-facilitative care staff communication behaviours are listed in descending order according to their frequency of use across all conversations

Abbreviations: CS–Care staff, CB–Communication Behaviour, PWD–Person with dementia, OR–Odds ratio, CI–Confidence interval

* significant, p < .05

** significant, p < .01

*** significant, p < .005

^(+)^ if the communication behaviour was used, the odds of content-based engagement were significantly increased

^(-)^ if the communication behaviour was used, the odds of content-based engagement were significantly decreased

#### Communication behaviours that reflect computer-identified CS referral to PWD content

**Fig 2:**
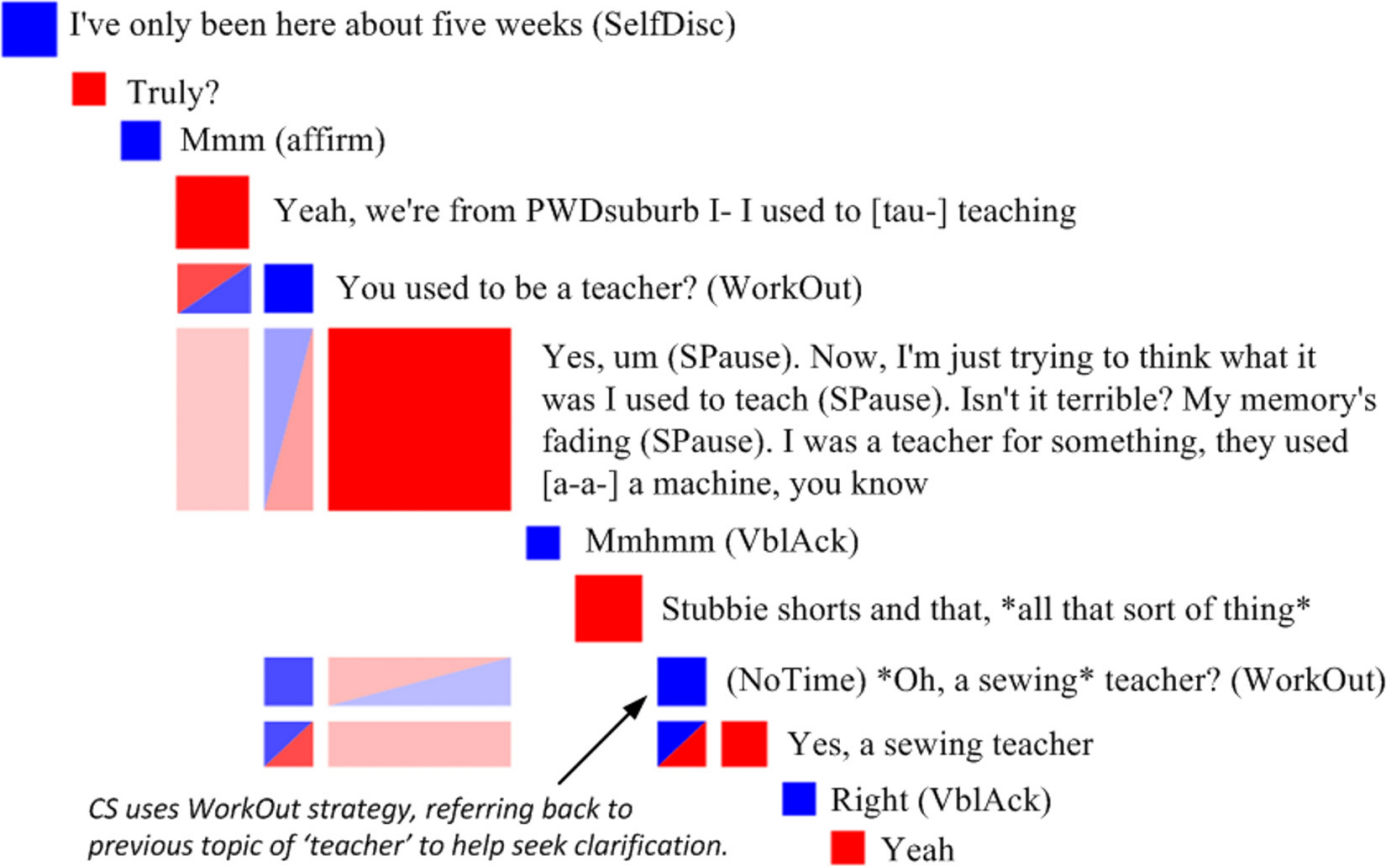
Zoom-in view of a Discursis plot shows how the coded communication behaviour ‘Work out’ aligns with backward recurrence where the care staff member (CS) used previous content provided by the person with dementia to work out what the person with dementia wants to say. Blue boxes along the diagonal represent care staff member turns. Red boxes along the diagonal represent turns by the person with dementia. Red-blue shaded boxes under the diagonal indicate instances of shared recurrence.

As can be derived from Table 4, the strongest link between human- and computer-generated coding of specific communication behaviours was found between ‘Active listening’ and backward short-term engagement of the CS with preceding PWD content (51%, *p* < .001, OR = 20.411). Another communication behaviour that reliably co-occurred with short-term engagement by the CS was ‘Visual topic’ (26%, *p* = .037, OR = 2.270). Communication behaviours that reliably involved CS backward medium-term engagement were ‘PWD Topic’ (36%, *p* < .001, OR = 11.056), ‘Work out’ (21%, *p* < .001, OR = 4.900), ‘Answer content’ (16%, *p* .001, OR = 3.446), ‘Rephrase’ (13%, *p* = .038, OR = 2.548) and ‘Give time’ (10%, *p* = .005, OR = 2.092). Long-term CS engagement by CS with PWD content was found for ‘PWD Topic’ (31%, *p* < .001, OR = 5.003), ‘Self disclosure’ (24%, *p* < .001, OR = 3.621), ‘Rephrase’ (19%, *p* = .013, OR = 2.563) and ‘Give time’ (12%, *p* = .046, OR = 1.594). A visualisation of the dynamics between CS communicative behaviour use (‘Work out’) and CS medium-term engagement with previous PWD content can be found in Fig 2.

#### Predictors of a lack of CS engagement

Similar to the results obtained in analysis 1, the use of ‘Verbal acknowledgment’ was found to be a significant predictor of a lack of engagement, in this case CS engagement with PWD content (5%, *p* < .001, .228). Additionally, it was found that CS use of ‘Active listening’ was associated with a lack of CS engagement with PWD content in the long-term (5%, *p* = .001, .500).

## Discussion

The current study applied a hybrid approach, combining human- and computer-generated labels of conversational utterances, in order to examine conversation dynamics between PWD and CS at residential aged care facilities. The first of the two aims of this study was to examine whether specific communication behaviours used by the CS are linked to subsequent content-based engagement in the conversation by the PWD. It was hypothesised that the spontaneous use of facilitative communication behaviours would result in an increased likelihood of content-based engagement by the person with dementia across subsequent turns.

### Communication behaviours linked to PWD engagement with CS content

**Fig 3:**
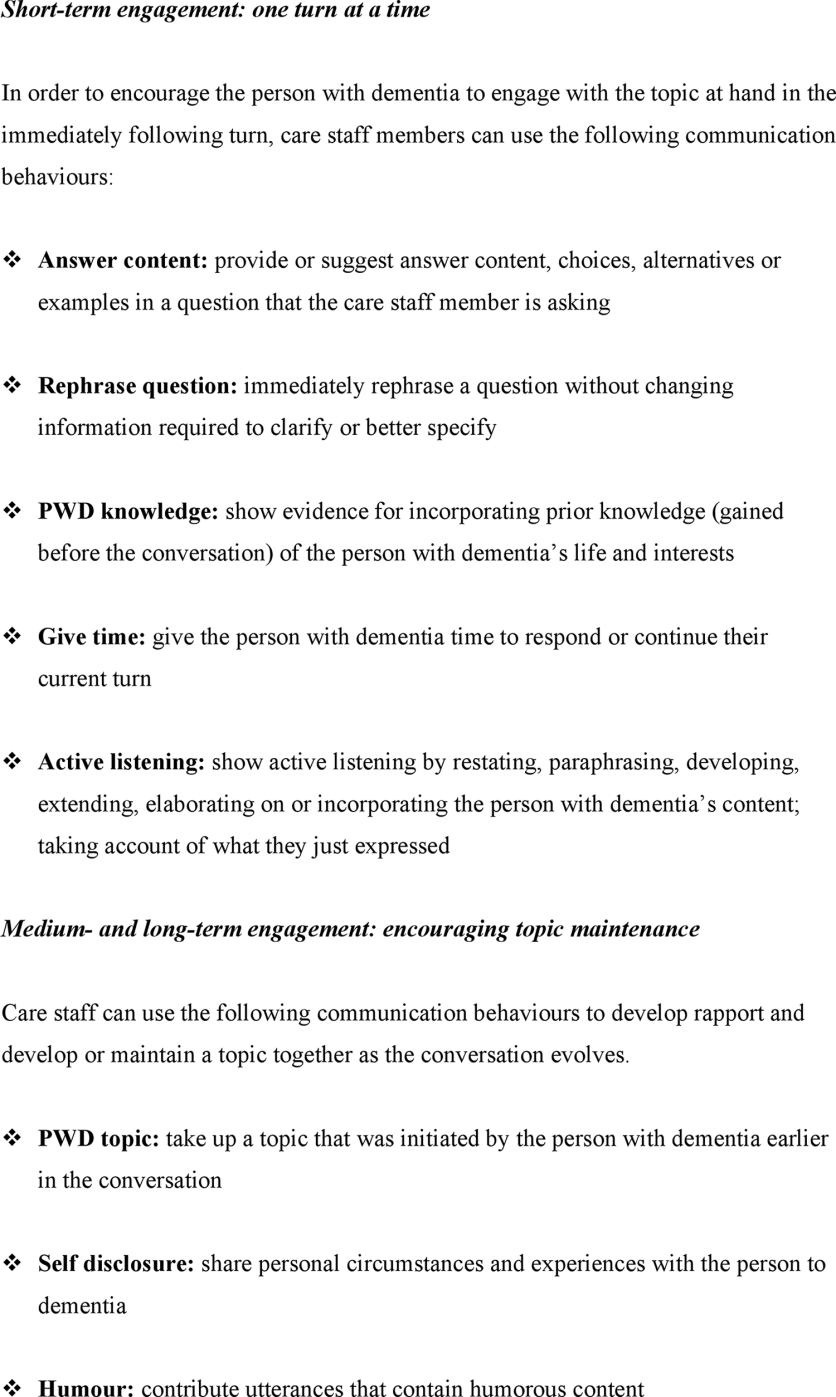
Overview of communication behaviours associated with different types of content-based engagement by the person with dementia.

A first series of analyses (analysis 1, see Table 3 and Fig 3) revealed that several communication behaviours outlined in the MESSAGE programme [21, 23] were indeed effective in eliciting content-based conversational engagement with people with dementia in the short-, medium- and long-term time-scales, following the turn in which the CS displayed the communication behaviour. As a reminder, short-term refers to utterances within one turn prior or subsequent to a current turn, while medium-term refers to utterances ten turns prior or subsequent to a current turn and long-term refers to all utterances prior or subsequent to a current turn. Out of the communication behaviours that were found to be effective in analysis 1, a distinction can be proposed between behaviours that emphasise the interpersonal relational aspects of conversation and compensatory behaviours that prompt for a choice, response or continuation of a narrative.

#### Interpersonal and individualised aspects of conversation

Previous qualitative research has established that warm interpersonal relationships are an important aspect of dementia care [42]. The current findings support and expand this assertion with quantitative results suggesting that communication behaviours that contribute to creating an interpersonal bond in a conversation increase the probability of the person with dementia’s content-based engagement in the conversation. Results indicated that when staff used communication behaviours that promote the interpersonal and individualized aspects of conversation, specifically, ‘Self disclosure’, ‘PWD knowledge’, ‘PWD topic’, ‘Humour’ and ‘Use name’, PWD tend to engage with content in the conversation. These communication behaviours reflect instances of the CS sharing information about CS’ own life, contributing information to the conversation that will be familiar to the PWD and reflecting prior knowledge about the PWD’s interests and habits, CS taking up topics initiated by the PWD, CS trying to keep the conversation good-humoured and CS using PWD’s first name.

If the CS drew upon his or her previous knowledge about the PWD to contribute to the conversation (‘PWD knowledge’), probabilities of PWD engagement with content from the CS utterance were 19% for short-term and 17% for medium-term engagement. If the CS referred to topics that the PWD mentioned earlier in the conversation (‘PWD topic’), a 19% probability of long-term engagement by the PWD with those CS utterances was found. This provides specific evidence to support suggestions that the use of familiar information or topics is beneficial for engaging a person with dementia [23, 37].

Sharing of personal details by the CS within a conversation (‘Self disclosure’) elicited medium- and long-term PWD engagement at probabilities of 13% and 22%, respectively. This finding suggests that when staff use content about their own lives, it encourages the PWD to engage back with this CS content throughout the remainder of the conversation or at least eleven utterances forward from the CS utterance where the self disclosure occurred. Humorous CS utterances (‘Humour’) were found to elicit subsequent long-term PWD engagement at a 19% probability, suggesting that humour could be successfully used to establish rapport.

Use of the person’s first name was associated with medium-term engagement, i.e. after hearing their name uttered in a staff member’s turn, there was a 16% probability that the PWD would share the content of that CS utterance anywhere across the subsequent ten turns. The increased occurrence of medium-term engagement likely reflects a successful orientation of the PWD’s attention to the conversation [21], which lasts throughout the medium-, but not the long-term. Therefore, it can be argued that, in addition to creating a personable atmosphere in a conversation, the CS use of the PWD’s name might also have provided a compensation for attention deficits [23].

Together, these findings comprise empirical evidence to support the concept that interpersonal/relational aspects of communication can assist with topic engagement. Overall, these results indicate that when care staff relate with the person using interpersonal and individualised communication behaviours in a conversation, the probability of PWD content-based engagement in the conversation is increased.

#### Effective support via direct and indirect prompts

In addition to the above-mentioned communication behaviours that focus on a warm, personal bond between conversation partners, behaviours that prompt the PWD for a choice, response or continuation of a narrative, directly (‘Answer content’, ‘Rephrase question’, Active listening’) or indirectly (‘Give time’) were also effective in eliciting PWD topic engagement. CS paraphrasing of PWD content (‘Active listening’) was found to elicit PWD engagement in the short-, medium- and long-term, indicating that the paraphrasing of previous PWD content by the CS might assist PWD with topic maintenance from one turn to the next, as well as within ten turns and throughout the remainder of the conversation. Behaviours that support PWD to respond to questions, including the provision of answer content within the question (‘Answer content’) by the CS, the rephrasing of questions (‘Rephrase question’) as well as the use of pauses after CS questions to permit sufficient time for a response (‘Give time’) was found to elicit PWD engagement within the subsequent turn (short-term). These results suggest the success of specific communication behaviours involving questions in supporting a content-based response from PWD.

These findings are congruent with previous reports of increased PWD responsiveness in interactions involving rephrasing and repetition by a conversation partner [43] and previous reports of the benefits of providing sufficient time for PWD to respond [44]. However, it is noteworthy that other behaviours involving rephrasing and clarification mechanisms, such as ‘Rephrase’ and ‘Work out’ were not found to be significant predictors of PWD engagement. This is likely to be linked to subtle differences in the focus of these communication behaviours. Specifically, that they seek to repair difficulties with understanding of the other person’s previous utterances either by PWD (‘Rephrase’) or CS (‘Work out’), and therefore has a backwards trajectory. While those question support behaviours are designed to support subsequent response utterances. This is reflected in findings of analysis 2.

#### Verbal acknowledgment: lack of content to share?

Another finding that was somewhat unexpected was the significant link between CS use of ‘Verbal acknowledgment’—a behaviour classified as facilitative—and an absence of PWD engagement with content from the CS turn where this behaviour occurred. However, the explanation of this link is likely to lie in the fact that, by definition, ‘verbal acknowledgment’ utterances lack referential meaning. Therefore these utterances would lack terms for Discursis to include in the computation of metrics, and content for PWD to take up in their subsequent utterances. (for an example, see CS turns in Fig 1). Any expansion in a CS utterance beyond non-lexical or phrasal back-channelling would have been coded as a behaviour other than ‘Verbal acknowledgment’, such as, for example, ‘Active listening’ or ‘Work out’, according to the communicative behaviour definitions (Table 1).

#### Summary of findings (analysis 1)

To summarise the discussion of analysis 1, it was found that nine behaviours out of the 22 total communication behaviours that are aligned with the MESSAGE strategies are associated with content-based engagement of the PWD, and one is associated with a lack of content-sharing. The current findings of short-term engagement in relation to behaviour involving rephrasing, paraphrasing, suggesting content, and provision of pauses is not surprising given the communication behaviours they represent. It is those behaviours that elicited medium- and long-term PWD engagement that are particularly noteworthy for their discourse-building effect during conversations with PWD.

Considering the prevalence of memory problems in dementia, which can impair the person’s skills in discourse topic maintenance [14, 45], it is of particular interest that the occurrence of medium- and long-term PWD engagement was found to be probable to this extent when ‘PWD knowledge’, ‘PWD topic’, ‘Self disclosure’, ‘Humour’, ‘Use name’ and ‘Active listening’ were used. An overview of communication behaviours that care staff members can use to encourage short-term and medium-/long-term content-based engagement is provided in Fig 3.

### Alignment of human- and computer-generated labelling

Analysis 2 aimed to investigate whether computer-generated output of instances of content-based engagement by the care staff reflect human-generated codings of specific facilitative behaviours. It was hypothesised that some communication behaviours would show alignment with Discursis-indicated content-repetition while other communication behaviours might entail dynamics that the Discursis metrics used in the current analysis cannot express. There are numerous communication behaviours that relate to a CS member’s ability to take up content that the PWD has previously used, in order to engage with the PWD, such as ‘Active listening’, ‘PWD topic’ and ‘Work out’. Therefore, it was hypothesised that a Discursis analysis would be able to capture the use of these behaviours through the CS-other-backward metrics.

#### Metrics are sensitive to differences in the time course of repetition reflected in human coded behaviours

Findings obtained in analysis 2 (see Table 4 and Fig 3) did indeed reveal a significant co-occurrence of Discursis-indicated shared backward recurrence and CS communication behaviour use characterised by elements of content uptake. These included ‘Active listening’, ‘PWD topic’ and ‘Work out’. Of particular interest is the success that Discursis-metrics had in differentiating between behaviours that are human coded due to immediate repetition (‘Active listening’), compared to communication behaviours coded for repetition of content used further back in the conversation (‘PWD topic’ and ‘Work out’).

Specifically, the human coded occurrences of ‘Active listening’ behaviour showed a 51% probability of also being marked as short-term CS-other-backward engagement. This finding was expected, considering that ‘Active listening’ is coded when the CS repeats or rephrases immediately preceding PWD content. In contrast, instances of repeating or rephrasing of utterance content several turns prior to a given CS utterance would have been coded by human raters as ‘PWD topic’ or ‘Work out’ (see Table 1). ‘PWD topic’ is defined as an instance where the CS refers back to content previously provided by the PWD in order to encourage PWD’s narrative. Findings reflect that use of this communication behaviour was indeed associated with a 36% probability of medium-term and a 31% probability of long-term CS engagement with previous PWD content. ‘Work out’ is defined as an instance where the CS refers back to PWD content to seek further clarification (see Fig 2). Occurrences of this communication behaviour were associated with a 21% probability of medium-term CS engagement with previous PWD content. The differences in findings of short- versus medium-/long-term engagement for these particular behaviours indicate that Discursis metrics have been sensitive to the differences in time course of repetition that is reflected in the human coded behaviours.

#### Reasons for incomplete overlap: term-based versus concept-based Discursis modes

Another aspect of these metrics that is of interest is the proportion of overlap. It may at first be expected that these communication behaviours that are defined as the uptake of content from the PWD would overlap with CS-other-backward metrics at close to 100%. This was not the case. ‘Active listening’ had the highest proportion of overlap at 51%, with ‘PWD topic’ at 36% and ‘Work out’ at 21%. A likely explanation for these levels of overlap would be related to the use of term-based mode in the current Discursis analysis.

Using the term-based analysis approach, CS-other-backward was only coded by Discursis if the if the CS repeated one or more of the exact words that were used by the PWD. Therefore, instances where the CS may still have taken up content from the PWD turn, but paraphrased the wording would not have been identified as term-based recurrence by Discursis. Interestingly, the pattern of decreasing proportion of direct term-based overlap between ‘Active listening’ and the other communication behaviours (‘PWD topic’ and ‘Work out’) found in the current analysis suggests that CS are more likely to use the same terms in immediate repetition, compared to recurrence in the longer term (‘PWD topic’) or when trying to paraphrase unclear information (‘Work out’).

The overlap of the Discursis and human coding occurrences may be increased in the future by replicating the current analysis in the concept-based mode of Discursis where semantic association is used to build synonymous groups of words that are recognised as recurrence. It is important to note, though, that the application of the term-based mode in the current analysis was purposefully chosen. Due to the relative paucity of overall terms and semantic concepts in the current conversations, the Discursis concept-based mode was deemed inappropriate because the conversation datasets lacked the semantic richness to create the internal language model that the concept-based mode relies on to compute recurrence of concepts.

#### Novel insights into the dynamics of the communication behaviours that are part of the MESSAGE programme

The current analysis also found that some communication behaviours that are not specifically linked with content uptake by the CS in their definition were still linked with CS-other-backwards Discursis metrics. This analysis has therefore led to consideration of the role of content uptake by the CS within the application of other communication behaviours that are recommended by the MESSAGE training programme.

Firstly, the ‘Visual topic’ communication behaviour was associated with short-term CS engagement with PWD content (26% probability). ‘Visual topic’ is marked when the CS is using photos and other visual cues to facilitate the interaction, but the behaviour is not specifically linked with either party initiating content. This finding might reflect that the use of the visual cue can be accompanied by PWD content, which is subsequently picked up by the CS in conjunction with referring to the visual cues in the room (e.g. photos on the wall), or the presence of the cue assists the CS to pick up the content from the PWD.

Another communication behaviour that is not defined by its relationship with PWD content but has been found to be associated with long-term CS engagement with previous PWD content is ‘Self disclosure’. This behaviour is marked when the CS member provides information about themselves or their own experiences. This finding highlights that CS may be relating their own experiences to content initiated by the PWD earlier in the conversation, which may be an across conversation behaviour for cohesion, or that by drawing upon information that the PWD has previously used in conversation the CS attempts to facilitate the comprehension of the PWD.

Per original definition, ‘Rephrase’ and ‘Answer content’ are not characterised by their relationship to PWD content, but by their relation to CS’ own content, i.e. CS self-repetition. ‘Rephrase’ was coded when the CS reworded his or her own utterance from a given CS turn to CS’ immediately subsequent turn in response to a lack of understanding or uptake from the PWD. ‘Answer content’ was marked when the CS suggests possible answers to CS’ own question within CS’ own turn, to assist the PWD with word-finding. Both communication behaviours were associated with medium-term CS engagement with previous PWD content and ‘Rephrase’ was also associated with long-term CS-other-backward engagement. The lack of association with short-term engagement suggests a sensitivity of Discursis to the definition of these behaviours, i.e. any short-term repetition around the occurrence of these communication behaviours would more likely be reflected in CS-self-backward, rather than CS-other-backward.

The findings of associated medium- and long-term CS-other-backward recurrence might suggest that CS members are referring back to previous content from the preceding ten (or more, for long) PWD turns when applying these behaviours. This finding may indicate a desire by the CS to build cohesive discourse and maintain topic across the conversation. It might also be that the CS are using broader strategies (not linked to individual utterances) by drawing upon information that the PWD has previously used in the conversation to facilitate comprehension of the PWD.

The final communication behaviour that was associated with CS engagement with PWD content was ‘Give time’ in the medium- and long-term. This finding was somewhat unexpected as the behaviour has a forward trajectory, wherein its influence is considered to be on the following utterance after the time is given. The ‘Give time’ behaviour is marked on utterances where the CS posed a question and was marked in the current dataset with a particularly high frequency (used 204 times, see Table 1 for frequencies of all communication behaviours within the current dataset), likely with a high degree of co-occurrence with other behaviours. It is suggested that these factors may lead to the ‘Give time’ behaviour often co-occurring on utterances with other behaviours that are more specifically linked with content sharing, and therefore with CS-other-backward metrics. Alternatively, it could be speculated that, as the ‘Give time’ is linked with medium- and long-term engagement, the provision of time to respond to questions allowed the PWD to produce more content in their turn, subsequently leading to the CS having more PWD-initiated content to engage with over the course of the conversation.

#### Verbal acknowledgment: lack of content to share?

Similar to the findings obtained in analysis 1, ‘Verbal acknowledgment’ was associated with a lack of engagement (short-term). The explanation of this result is likely to be the same: turns that contain non-verbal back-channelling utterances would lack terms for Discursis to include in the computation of metrics. If the CS was merely providing a verbal acknowledgment in a given turn without interrupting the PWD’s narrative, it is likely that this CS turn would not share PWD content.

#### Summary of findings (analysis 2)

In summary, analysis 2 revealed that Discursis was successful in identifying communication behaviours that are characterised by CS uptake of PWD content, and was sensitive to the time course differences between these behaviours. The findings also reveal that further consideration is required for the role of content uptake for some behaviours that are not primarily defined by the role of CS engaging with PWD content. Specifically, CS might rely on behaviours that involve using recurring topic themes across the time course of the conversation to facilitate topic cohesion, or comprehension of content by the PWD.

### Limitations and future directions

The current analysis is limited to the assessment of term-based sharing between speakers, which cannot encapsulate other important conversation parameters, such as voice tone, eye contact, physical contact, facial expression and gestures, which are all additional important contributors to conversations between people with dementia and their conversation partners [43, 46–51]. Furthermore, the analysis performed in this study included one-off baseline conversations, without information on changes in CS and PWD communication behaviours post-training of the CS with the MESSAGE programme. Finally, the short and accidental interruptions by others might potentially have unnecessarily disrupted the conversations, affecting topic maintenance, which could have been avoided by recording in a more secluded environment.

The approach presented here can assist in automatically generating novel and crucial feedback for carers in any setting where training and ongoing assessment of communication skills is desirable. For example, transcripts of training sessions where carers are engaging in conversations with a given patient group of interest (or an actor, hired to simulate a given condition) could be processed in Discursis to provide objective visual and quantitative feedback on conversation parameters of interest. Discursis plots and metrics output are also well-suited in any settings where it would be helpful to keep track of progress in communicative abilities over time.

Following the current provision of an approach to empirically measure communication dynamics in relation to communication behaviour use, a future focus on pre- and post-training dynamics might provide further insights into the efficiency of each of these communication behaviours in facilitating content-based engagement as well as response to training in individual trainees. Finally, the Discursis metrics assessed were the primitive Discursis metrics only. These metrics might not capture certain conversation behaviours as adequately as more tailored metrics, which focus on recurring motifs within a conversation rather than simple backward and forward recurrence. Therefore, a future focus on the creation of tailored Discursis metrics that more specifically reflect conversation behaviours relevant to the MESSAGE training programme might be beneficial.

## Conclusion

The current study demonstrates the added merit and limitations of a computer-assisted text analysis tool in assessing content-based conversational engagement in health care settings. First, communication behaviours that enhance content-based engagement by people with dementia could be identified, including those that support the interpersonal relational aspects of conversation and those that compensate for linguistic impairments associated with dementia. Second, Discursis was found to reliably align with several human codings of communication behaviour. Finally, the current analysis sparked a re-consideration of the role of content sharing for some of the communication behaviours that are part of the MESSAGE communication training programme for carers of people with dementia.
